# The emerging roles of long non‐coding RNAs in polyglutamine diseases

**DOI:** 10.1111/jcmm.16808

**Published:** 2021-07-28

**Authors:** Xiaoyu Dong, Shuyan Cong

**Affiliations:** ^1^ Department of Neurology Shengjing Hospital of China Medical University Shenyang China

**Keywords:** biomarker, long non‐coding RNA, neurodegeneration, polyglutamine diseases, therapy

## Abstract

Polyglutamine (polyQ) diseases are characterized by trinucleotide repeat amplifications within genes, thus resulting in the formation of polyQ peptides, selective neuronal degeneration and possibly death due to neurodegenerative diseases (NDDs). Long non‐coding RNAs (lncRNAs), which exceed 200 nucleotides in length, have been shown to play important roles in several pathological processes of NDDs, including polyQ diseases. Some lncRNAs have been consistently identified to be specific to polyQ diseases, and circulating lncRNAs are among the most promising novel candidates in the search for non‐invasive biomarkers for the diagnosis and prognosis of polyQ diseases. In this review, we describe the emerging roles of lncRNAs in polyQ diseases and provide an overview of the general biology of lncRNAs, their implications in pathophysiology and their potential roles as future biomarkers and applications for therapy.

## 1 INTRODUCTION

Polyglutamine (polyQ) diseases are a group of common neurodegenerative diseases caused by the abnormal repetitive amplification of CAG in the coding region of each pathogenic gene and the formation of polyQ peptides, thus resulting in selective neuronal degeneration and death in neurodegenerative diseases^1^. To date, nine types of polyQ diseases have been found: Huntington's disease (HD), spinobulbar muscular atrophy (SBMA), dentatorubral‐pallidoluysian atrophy, spinocerebellar ataxia type 1 (SCA1), SCA2, SCA3, SCA6, SCA7 and SCA17.^2^ Most patients with polyQ disease show adult onset, which is mainly characterized by progressive neurological dysfunction. In addition to spinobulbar muscular atrophy, the other eight polyQ diseases show an age of onset and severity associated with the length of CAG repeat amplification.^3^ At present, the pathogenesis of polyQ diseases is unclear, and hypotheses regarding possible routes of pathogenesis include abnormal polyQ protein aggregation and formation of nuclear inclusion bodies, abnormal transcriptional regulation, interactions among pathogenic proteins, RNA toxicity, abnormal protein modification and apoptosis.

With the development of high‐throughput sequencing technology, many lncRNAs have been discovered, and a preliminary understanding of the functions and mechanisms of these lncRNAs has been gained.^4^ LncRNAs regulate gene expression and act as signalling molecules, protein complex scaffolds and molecular baits to achieve their biological functions.^5^ LncRNAs have been shown to be involved in the pathogenesis and progression of a variety of neurodegenerative diseases, including polyQ disease.^6^ Some studies have also shown that an increase or decrease in lncRNA expression can serve as potential diagnostic biomarkers, improve neurodegenerative processes and promote endogenous regeneration.^7^

In this review, we briefly introduce typical lncRNA biogenesis and functions, and we describe the most relevant lncRNAs specifically associated with polyQ disease. The advantages and limitations of potential biomarkers involved in the diagnosis and prognosis of in polyQ disease, as well as the use of lncRNA‐based therapeutic strategies, are also highlighted.

## 2 BASICS OF LNCRNAS

LncRNAs are non‐coding RNAs that are structurally similar to messenger RNAs, but lack an open reading frame and are longer than 200 base pairs. LncRNAs are transcriptional products of RNA polymerase II and are distributed in the nucleus and cytoplasm.^8^ In 2002, Schrauwen,^9^ a Japanese researcher, first discovered and identified a long transcription product when sequencing a mouse DNA library and named it lncRNA. Recent studies have shown that although lncRNAs do not encode proteins, they are involved in DNA methylation, nucleolar dominance, X chromosome silencing, genomic imprinting and chromatin modification, transcriptional activation and regulation, RNA interference, intranuclear transport and other important regulatory processes.^10^, ^11^, ^12^ Although most lncRNA sequences have only a low degree of evolutionary fidelity, a small number of sequences have been conserved among various species. LncRNAs are believed to have arisen from the following sources: (1) a lncRNA incorporating the precursor sequence of a coding protein gene can be formed by breaking the protein‐coding gene; (2) a lncRNA containing multiple exons can be reconstructed from two unrelated sequences and one separated sequence; (3) functional or non‐functional ncRNA can be produced by reverse transcriptional replication of non‐coding genes; (4) lncRNAs can be formed by insertion of transposons; and (5) lncRNAs can be formed by tandem replication of adjacent replicators.^13^

**FIGURE 1:**
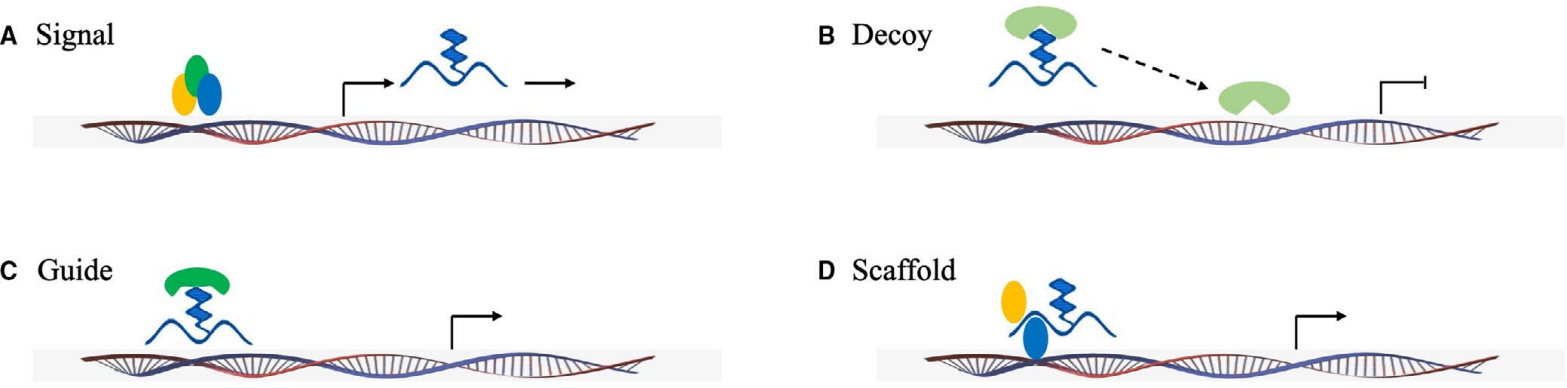
The roles and molecular mechanisms of lncRNAs. (A) As signals, lncRNA expression can reflect the combinatorial actions of transcription factors (coloured ovals) or signalling pathways, and indicate gene regulation. (B) As decoys, lncRNAs can competitively bind transcription factors and other proteins and sequester them from chromatin. (C) As guides, lncRNAs can recruit chromatin‐modifying enzymes to target genes; (D) As scaffolds, lncRNAs can bring multiple proteins into proximity to form ribonucleoprotein complexes

According to the relative positions of the coding sequence of the lncRNA and the protein‐coding gene, lncRNAs can be divided into the following categories: (1) sense lncRNAs overlapping with the sense strand of the protein‐coding sequence; (2) antisense lncRNAs overlapping with the antisense strands of protein‐coding sequences; (3) bidirectional lncRNA sequences located on the antisense strand, at a distance more than 1000 bp from the transcription start site, with the two directions of transcription being opposite; (4) intron lncRNA sequences located completely in the intron region of another transcript; and (5) intergenic lncRNA sequences, which are not adjacent to any protein‐coding gene and originate from the gene spacer between two protein‐coding genes.^14^ According to their molecular mechanisms and roles, lncRNAs can be divided into signal molecules, decoy molecules, guide molecules and scaffold molecules (Figure 1). LncRNAs are believed to regulate gene expression at three levels: the epigenetic modification level, transcriptional level and post‐transcriptional level. (1) In epigenetic level regulation, lncRNAs regulate gene expression through processes including DNA methylation or demethylation, RNA interference, histone modification and chromosome remodelling. For example, the lncRNA HOTAIR induces heterochromatin formation at specific gene loci through interaction with the nuclear chromatin remodelling complex, thereby decreasing the expression of a target gene.^15^ (2) In regulation at the transcriptional level, lncRNAs regulate the expression of target genes by recruiting transcriptional regulators to promoters adjacent to target genes. LncRNAs participate in genome regulation at the transcriptional level in a variety of ways.^16^ (3) In post‐transcriptional regulation, lncRNAs form RNA dimers with target RNA through complementary base pairing, thus hindering the binding of transcription factors or related RNA processing factors, or directly recruiting translation inhibitor proteins, thereby regulating the splicing, translation and degradation of RNA.^17^

The development of the central nervous system (CNS) requires precise expression and regulation of specific genes in time and space. Many factors, including genetic and environmental factors, affect the development of CNS and can lead to a series of neurological diseases. Studies have shown that lncRNAs are abundantly distributed in the CNS, presumably because the complexity of the brain requires many regulatory RNAs to maintain normal development and function, including brain development, neuronal differentiation and maintenance, synaptic plasticity, cognitive function and learning and memory processes.^18^ Recent studies have shown that lncRNAs are abnormally expressed in older people and in neurological disease states, thus suggesting that lncRNAs may regulate the occurrence and development of neurological diseases. The large number and tissue‐specific expression of lncRNAs make them potential biomarkers for disease diagnosis and prognosis. Feng et al. have indicated that the LncRNA BACE1 (95% CI: 0.553–0.781, *p* = 0.003) is elevated in the plasma in patients with Alzheimer's disease (AD) and has high specificity (88%) for AD; therefore, LncRNA BACE1 may be a potential candidate biomarker for predicting AD.^19^ Hossein‐Nezhad et al. have studied the cerebrospinal fluid of patients with Parkinson's disease and found two differentially expressed lncRNAs, UC001 lva.4 (*p*  =  0.01, log_2_FC  =  –1.6) and AC079630 (*p*  =  0.001, log_2_FC  =  –6.72), which are significantly down‐regulated and might be used for early prediction and detection of Parkinson's disease.^20^ By studying the expression of lncRNA in the nervous system in patients with HD, Johnson et al. have found that LINC0341, TUG1 and RPS20P22 are up‐regulated in HD, whereas LINC00342 is down‐regulated.^21^ These studies have shown the potential of lncRNA to serve as a molecular biomarker for the diagnosis of CNS diseases.

## 3 LNCRNAS IN POLYQ DISEASE

### 3.1 LncRNAs in HD

**FIGURE 2:**
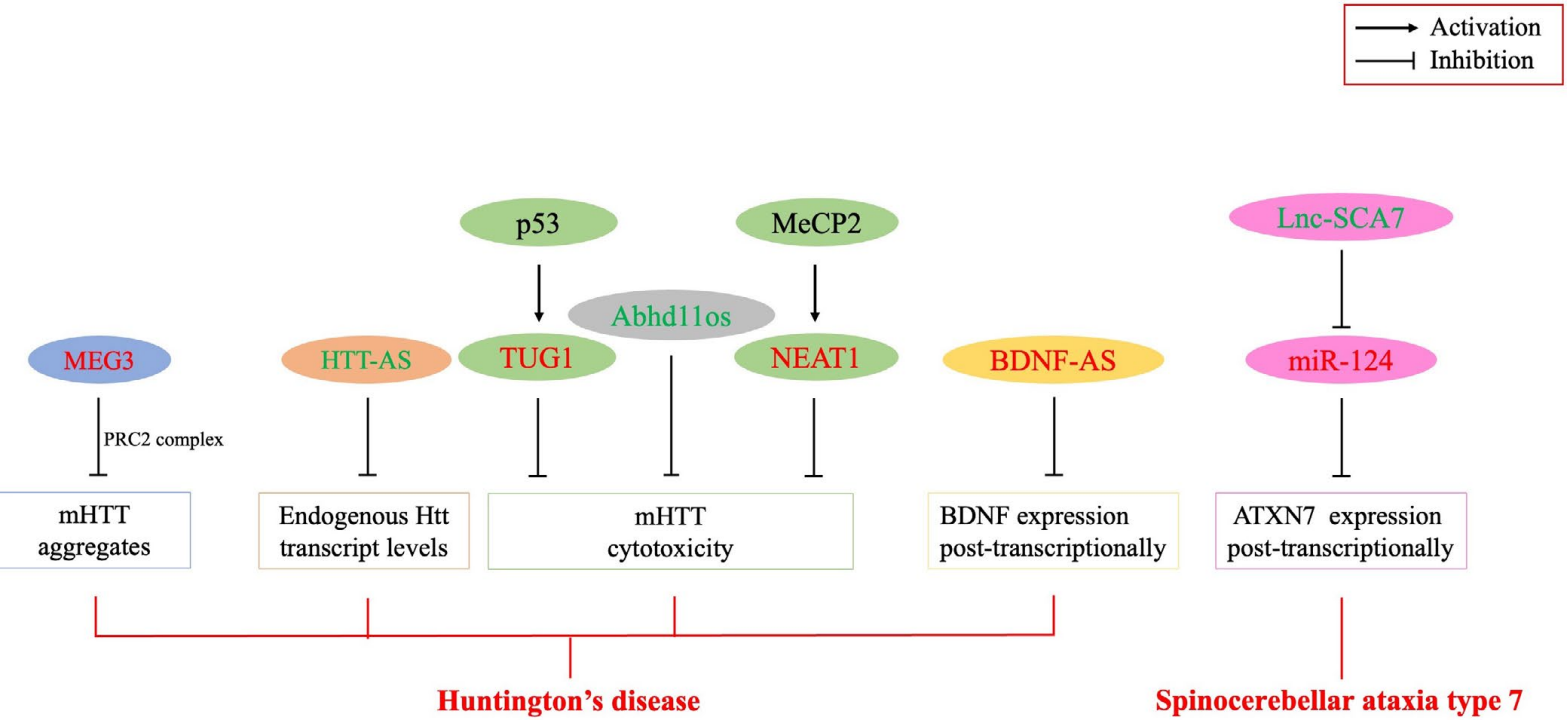
A summary of the most dysregulated lncRNAs in polyQ disease. HD/SCA7‐related lncRNAs are grouped according to the pathogenesis in which they have been implicated. PRC2, polycomb repressive complex 2; MeCP2, methyl‐CpG‐binding protein 2

The prevalence of HD in Europe and North America is 5–10 per 100,000 people.^22^ CAG trinucleotide duplication in the *Huntington* gene leads to abnormal accumulation of misfolded Huntington protein (HTT) in nuclear inclusion bodies and progressive loss of striatal neurons, which are the main pathogenic factors of the disease.^23^ The clinical features of HD are chorea, dystonia and cognitive or mental disorders.^24^ Altered levels of lncRNAs have been found to contribute to the dysregulation of genes observed in HD and to modulate HD pathogenesis. We will review some of the consistently identified as dysregulated lncRNAs associated with HD pathology in the following section (Figure 2; Table 1).

**TABLE 1:** Dysregulated lncRNAs in Huntington’ disease

Official symbol	Genomic location	Roles of lncRNAs	Expression level	References
HAR1	3	Aberrant nuclear‐cytoplasmic REST trafficking caused by mutated huntingtin resulting the aberrant expression of HAR1 in striatum	Down‐regulated in the brain	[25, 26]
TUNA	14	Significantly associated with the severity of pathological HD and decreased with increasing disease severity	Down‐regulated in the brain	[30]
NEAT1	11q13.1	Involved in the neuroprotective mechanism of alleviating mHTT‐induced toxicity, modulated by MeCP2	Up‐regulated in the brain	[33, 34]
MEG3	14q32	It associates with PRC2 complex, and modulates the formation of aggregates of mHTT	Up‐regulated in the brain	[35, 36]
Abhd11os	5; 5	Abhd11os overexpression produces neuroprotection against the neurotoxicity of mHTT	Down‐regulated in the brain	[35]
HTT‐AS	4p16.3	HTT‐AS decreases endogenous HTT transcript levels	Down‐regulated in frontal cortex	[39]
DGCR5	22q11	Downstream target of REST in HD	Down‐regulated in the brain	[41]
TUG1	22q12.2	Target of p53, up‐regulation has the function of antagonizing mHTT cytotoxicity	Up‐regulated in the brain	[42]
BDNF‐AS	11p14.1	Decreasing BDNF expression post‐transcriptionally	Up‐regulated in the brain	[54]

Human accelerated region 1 (HAR1) is a segment of the human genome found on the long arm of chromosome 20, a highly conserved genomic region consisting of a *cis*‐antisense pair of structured lncRNAs (HAR1F and HAR1R) specifically transcribed in the nervous system.^25^, ^26^ Johnson et al., through autopsy studies, have found that HAR1F and HAR1R levels are significantly diminished in the striatum in patients with HD, whereas the levels in the cerebral cortex show no significant changes. Moreover, the authors have also confirmed that HAR1 is a direct target of RE1‐silencing transcription factor (REST), which plays a critical role in the pathogenesis of HD. This targeting is likely to cause both forward and reverse HAR1 transcripts to be down‐regulated in the striatum in patients with HD.^27^

TCL1 upstream neural differentiation‐associated RNA (TUNA) is a highly conserved sequence in vertebrates and is specifically expressed in the CNS in mice and humans.^28^ Regulation of TUNA expression in mouse embryonic stem cells affects global gene expression, which is highly involved in cell differentiation, cell death and neurogenesis.^29^ TUNA forms an RNA‐multiprotein complex that is enriched at the promoters of Sox2, Nanog and Fgf4. Lin et al. have suggested that TUNA expression declines in brain samples in patients with HD, particularly in the thalamus and striatum. Furthermore, by retrieving data from a gene expression study on 44 patients with HD and 36 controls,^30^ the authors have found that the expression of TUNA is significantly associated with the severity of pathological HD and significantly decreases with increasing disease severity. Interestingly, this phenomenon was evident only in the striatum, whereas no significant changes were observed in the motor cortex and cerebellum.^31^

Nuclear paraspeckle assembly transcript 1 (NEAT1) is transcribed by RNA polymerase II into two different subtypes, NEAT1S and NEAT1L, which are short subtypes and long subtypes, respectively.^21^, ^32^ Sunwoo et al. have validated the increased NEAT1S levels in the R6/2 mouse brain as well as the post‐mortem brains of humans with HD by quantitative PCR analysis. Their results have further confirmed that up‐regulation of NEAT1S is involved in the neuroprotective mechanism against anti‐neuronal injury, rather than the pathological process of neurodegenerative changes in patients with HD.^33^ Cheng et al. have found that NEAT1L is also significantly elevated in striatum neurons of the brain in mice and patients with HD. With knockout of mHTT in vitro and in vivo, NEAT1L returns to normal levels; thus, the increase is mHTT‐dependent. The authors have also indicated that this dysregulation is associated with methyl‐CpG‐binding protein 2 (MeCP2), which interacts with NEAT1L directly or indirectly. Moreover, like Sunwoo et al., Cheng et al. reached the same conclusion that NEAT1L has a protective role in cells, which may help alleviate mHTT‐induced toxicity.^34^

Maternally expressed gene 3 (MEG3) is expressed in many normal human tissues, with the highest expression in the pituitary followed by various regions of the brain.^21^ Francelle et al. have reported that MEG3 levels are diminished in HD by mining microarray data; however, in cellular and R6/2 mouse models, the levels of MEG3 have been validated to be increased.^35^ Chanda et al. have further confirmed that the levels of MEG3 are significantly increased in cell and animal models, and MEG3 modulates the formation of aggregates of mHTT. Knockdown of MEG3 in an HD cell model significantly decreases the aggregates formed by the mHTT and the down‐regulation of endogenous tp53 expression.^36^

Abhd11os (called ABHD11‐AS1 in humans) is a putative lncRNA whose expression is enriched in the mouse striatum.^37^ Francelle et al. have demonstrated that Abhd11os is significantly decreased in animal models of HD. Moreover, artificial overexpression of Abhd11os decreases the neurotoxicity of mHTT, whereas Abhd11os knockdown exacerbates mHTT toxicity, thus indicating the significance of Abhd11os in HD.^35^

Huntingtin antisense (HTT‐AS) is a natural antisense transcript at the HD repeat locus, which forms a 5′ head‐to‐head divergent pair overlapping with the CAG expansion region and the 5´ UTR of HTT mRNA.^38^ Zucchelli et al. have confirmed the expression of HTT‐AS in the brain and implicated its participation in neuronal differentiation.^39^ HTT‐AS v1 (exons 1 and 3) is down‐regulated in the human HD frontal cortex; however, its function remains unknown.^40^

Previous studies have reported that other lncRNAs may be involved in the pathogenesis of HD. DiGeorge syndrome critical region gene 5 (DGCR5) is a neurospecific disease‐associated transcript that may play an important role in the human nervous system.^41^ It has been reported to be down‐regulated in HD; however, no functional studies have been performed on DGCR5.^40^ Taurate up‐regulated gene 1 (TUG1) is highly expressed in the mammalian brain and was originally found in a genome screen for genes up‐regulated after taurine treatment of developing retinal cells.^42^ It has been reported to be a target of p53 and to be up‐regulated in patients with HD.^43^ This up‐regulation, possibly induced by p53 activation, may antagonize mHTT cytotoxicity.^21^

### 3.2 LncRNAs in SCAs

SCAs are a complex group of fatal neurodegenerative diseases that primarily affect the brainstem, cerebellum and spinal cerebellar tract.^44^ Of the more than 40 SCA types, at least six (SCA1, SCA2, SCA3, SCA6, SCA7 and SCA17) are associated with polyQ disease.^45^ They are clinically characterized by gait and limb ataxia, dysarthria and abnormal eye movements. SCAs usually develop in adulthood and exhibit significant clinical heterogeneity. Symptoms usually appear between the ages of 30 and 40 and progress slowly.^46^ The size of the mutant allele CAG amplification is inversely correlated with the age of onset, and this phenomenon is more pronounced in patients with SCA2 and SCA7.^47^ Mutations in different types of SCA have been identified in different regions of the genome, and several involved genes have been mapped and cloned. Increasing evidence suggests that these diseases have the same molecular mechanisms and pathophysiological processes as other neurodegenerative diseases. Most SCA mutations involve the expansion of the trinuclear CAG sequence, which encodes a polyglutamine tract.^48^

However, only a few studies have confirmed the differential expression of some lncRNAs in SCAs. NEAT1L is not only dysregulated in patients with HD but also highly expressed in the SCA1, SCA2 and SCA7 mouse brain.^34^ The significance of the elevated expression of NEAT1L in SCA has not been verified experimentally, but given previous conclusions in HD studies, we infer that NEAT1L may play a protective role in the setting of CAG repeat expansion disease. Another notable study has examined SCA7, a neurodegenerative disease caused by repeated amplification of CAG in ATXN7 (which encodes a basic component of the mammalian transcriptional synergistic activation complex, STAGA), although the factors underlying the characteristic progressive cerebellar and retinal degeneration in patients are unclear. Lnc‐SCA7 arises from retrotransposition of the gene encoding ataxin‐7‐like protein 3 (Atxn7l3), a distant paralog of Atxn7, and the expression of lnc‐SCA7 has been found to be significantly associated with that of ATXN7 across human and mouse adult tissues and postnatal CNS regions. Through the study of an SCA7 mouse model, Tan et al. have found that lnc‐SCA7 modulates the expression of Atxn7 via a transcript‐dependent mechanism, which is likely to be achieved through the miR‐124 expression level rather than the translation of its putative ORF.^49^

## 4 LNCRNAS IN THE DIAGNOSIS AND TREATMENT OF POLYQ DISEASE

The large number and tissue‐specific expression of lncRNAs, as compared with coding genes, make them possible markers for disease diagnosis and treatment.^49^ The lncRNA HTT‐AS can be detected in the blood in patients with HD and thus may have potential applications in molecular diagnosis.^38^ Brain‐derived neurotrophic factor (BDNF) belongs to a class of secreted growth factors that are essential for neuronal maturation and survival.^50^ BDNF‐AS, an overlapping antisense lncRNA, has been reported to inhibit expression of BDNF at the post‐transcriptional level.^51^, ^52^ The level of BDNF is diminished in the brain in patients with HD, and overexpression of BDNF in the forebrain in a mouse model has been confirmed to rescue the HD phenotype.^53^ Given that BDNF plays such a key role in HD, increasing BDNF levels by down‐regulating BDNF‐AS may be a reasonable method for HD treatment.^54^ HTT‐AS may be also a promising lncRNA for treating polyQ diseases. It forms 5´ head‐to‐head bifurcation pairs that overlap with the CAG amplification region and the 5´ UTR of HTT mRNA, thus regulating expression of the *HTT* gene.^39^ Gene therapy with lentiviral vectors has become an effective method for the treatment of hereditary diseases. LncRNAs and their loci can be targeted in treatments through the design and synthesis of specific nucleic acid sequences, such as CRISPR/Cas9 sequences, antisense oligonucleotides and small interfering RNAs. However, unlike mRNAs, most lncRNAs are located in the nucleus and have high‐level structures. Oligonucleotide drugs must enter the cell and bind their target RNAs to be effective, thus posing challenges in drug delivery and intrinsic affinity. To solve these problems, commonly used methods include modifying oligonucleotide sequences and developing nano‐drugs to improve drug delivery.^55^, ^56^, ^57^ Lentiviral vectors are another choice as a carrier of lncRNAs. Francelle et al., through in vivo experiments using lentiviral vector bearing Abhd11°s sequences in HD mice, have found that overexpression of Abhd11°s exerts a neuroprotective effect against an N‐terminal fragment of *mHTT*.^35^

Although the application of lncRNAs as diagnostic biomarkers and potential treatment strategies for polyQ disease has a bright future, many difficulties remain to be overcome before clinical application. Currently, the detection of circulating lncRNA faces several challenges. For example, a consensus is lacking regarding the reference genes of circulating lncRNAs; moreover, it is not possible to determine which genes are stable and can serve as internal reference genes, and how to use appropriate reference genes to calculate the expression of circulating lncRNA. Therefore, methods to improve the accuracy of detection must be further studied. Furthermore, differentially expressed circulating lncRNAs lack specificity for specific neurodegenerative diseases. For example, NEAT1 has been found to be differentially expressed in AD,^58^ Parkinson's disease^59^ and amyotrophic lateral sclerosis.^60^ The occurrence and development of polyQ disease is a result of the combined actions of multiple genes. Therefore, the detection of only one type of circulating lncRNA has limited specificity and sensitivity. Combined detection of multiple lncRNAs and the combined diagnostic application with traditional serum markers can greatly improve the diagnostic value and will be an important direction in future developments. The actual mechanism of lncRNAs as a therapeutic strategy is not fully understood. The development of Genasense failed because of the lack of in‐depth understanding of its mechanisms, thus revealing the importance of understanding mechanisms in drug development.^61^ Second, owing to the low conservatism of lncRNAs, some lncRNAs are expressed only in primates; therefore, establishing a general experimental model is difficult.^62^ For most lncRNAs, appropriate animal models have not yet been constructed, but the availability of such models will be essential to understanding lncRNA function. Third, although some experiments on the application of lncRNA have been performed, the experimental results are not very reliable because of the small sample sizes.^57^ However, with the gradual advancement of lncRNA research, the prospects of using lncRNAs for the treatment of polyQ disease are broad.

## 5 CONCLUSIONS

In recent years, researchers have gradually deepened understanding of lncRNA and have found that lncRNAs play roles in physiological and pathological processes through epigenetic modification, post‐transcriptional regulation, translation and post‐translational modification. Similarly, lncRNAs also play important roles in the pathogenesis of polyQ diseases. Because in vitro and in vivo studies have demonstrated significant effects on the inhibition of mutant proteins in polyQ diseases, the development of efficient lncRNA delivery technology should be a promising strategy in this direction. By exploring advanced molecular biology techniques, lncRNA‐mediated gene regulation may be a potential method for the treatment of polyQ diseases.

## CONFLICTS OF INTEREST

The authors declare that they have no conflicts of interest.

## AUTHOR CONTRIBUTION

**xiaoyu dong:** Writing‐original draft (equal). **Shuyan Cong:** Funding acquisition (lead); Resources (lead); Supervision (lead); Writing‐original draft (equal).

## Data Availability

Data sharing is not applicable to this article, as no new data were created or analysed in this study.
